# A case report of successful rescue using veno-arterial extracorporeal membrane oxygenation: managing cerebral-cardiac syndrome

**DOI:** 10.3389/fcvm.2024.1370696

**Published:** 2024-04-11

**Authors:** Zheng Wang, Qi-Feng Zhang, Miao Guo, Xiao-Xia Qi, Xiao-Hui Xing, Gang Li, Shuang-Long Zhang

**Affiliations:** Department of Critical Care Medicine, Peking University International Hospital, Beijing, China

**Keywords:** cardiac arrest, cardiogenic shock, cerebral-cardiac syndrome, cerebral infarction, circulatory failure, VA-ECMO, case report

## Abstract

**Introduction:**

The presence of cerebral-cardiac syndrome, wherein brain diseases coincide with heart dysfunction, significantly impacts patient prognosis. In severe instances, circulatory failure may ensue, posing a life-threatening scenario necessitating immediate life support measures, particularly effective circulatory support methods. The application of extracorporeal membrane oxygenation (ECMO) is extensively employed as a valuable modality for delivering circulatory and respiratory support in the care of individuals experiencing life-threatening circulatory and respiratory failure. This approach facilitates a critical temporal window for subsequent interventions. Consequently, ECMO has emerged as a potentially effective life support modality for patients experiencing severe circulatory failure in the context of cerebral-cardiac syndrome. However, the existing literature on this field of study remains limited.

**Case description:**

In this paper, we present a case study of a patient experiencing a critical cerebral-cardiac syndrome. The individual successfully underwent veno-arterial-ECMO (VA-ECMO) therapy, and the patient not only survived, but also received rehabilitation treatment, demonstrating its efficacy as a life support intervention.

**Conclusion:**

VA-ECMO could potentially serve as an efficacious life support modality for individuals experiencing severe circulatory failure attributable to cerebral-cardiac syndrome.

## Introduction

Craniocerebral disorders frequently coincide with cardiac impairment, manifested by anomalies in electrocardiographic patterns, heart rhythm irregularities, and alterations in cardiac enzyme levels. In more severe instances, the onset of cardiac arrest can pose a life-threatening risk (1, 2). Scholars have designated this phenomenon as cerebral-cardiac syndrome (CCS). Previous studies have shown that there is no effective treatment plan for CCS at present. The main strategy is still to treat primary brain diseases, and actively give basic treatments such as bed rest, ECG monitoring, oxygen inhalation, maintaining water electrolyte balance, anticoagulation, antiplatelet, reducing intracranial pressure, anti-infection, etc. (2). In addition, for patients with hypertension, hyperlipidemia, diabetes and other basic diseases, actively carrying out the treatment of combined diseases can effectively prevent the occurrence of CCS. For cardiogenic shock caused by life-threatening CCS, the primary task is to effectively maintain the patient's circulatory and respiratory function, so as to maintain the patient's life, but the previous basic treatment is difficult to quickly and effectively improve the patient's circulatory and respiratory function.

Extracorporeal membrane oxygenation (ECMO) technology has progressively assumed a pivotal role as a cardiopulmonary support modality for critically ill patients. In recent years, its utilization has expanded considerably in individuals requiring cardiopulmonary support. Notably, veno-arterial-ECMO (VA-ECMO) has gained widespread acceptance in patients experiencing circulatory failure, particularly those with refractory cardiogenic shock who are unresponsive to conventional support methods, thereby affording a temporal window for the targeted treatment of the underlying disease (3). Consequently, ECMO has emerged as a viable life support strategy for managing severe, life-threatening circulatory failure stemming from cardiogenic shock. There have been previous reports of ECMO as life support applied to neurogenic pulmonary edema secondary to craniocerebral disease, and even reports of successful application in hemorrhagic disease (4, 5), but it has not been found to be applied in patients with CCS. ECMO may be an effective means of life support for circulatory failure caused by serious life-threatening CCS.

In this case, the patient experienced cardiac arrest. Although conventional cardiopulmonary resuscitation initially led to partial restoration of spontaneous circulation, standard therapeutic approaches proved insufficient in sustaining optimal and effective circulation. Consequently, the VA-ECMO team was summoned for rescue intervention. Imaging conducted during the rescue operation revealed a substantial cerebral infarction, indicative of circulatory failure attributed to cerebral-cardiac syndrome. Following successful intervention, the patient was subsequently transferred to the rehabilitation department for further rehabilitation therapy.

## Medical records

The patient was a 40-year-old male with a weight of 100 kg and a height of 175 cm, no previous adverse life history, no family genetic history, usually in good health. He was hospitalized with the primary complaint of chest tightness persisting for 3 days and a 4-h episode of altered consciousness occurring 3 years after undergoing percutaneous coronary intervention (PCI) for acute myocardial infarction. The patient had a history of coronary angiography and PCI, during which a stent was placed due to 90% stenosis of the proximal right coronary artery. Post-surgery, the patient received oral secondary prevention drugs for coronary heart disease. A follow-up coronary angiography conducted 1 year prior indicated no in-stent stenosis. However, the patient had not consistently taken anti-platelet aggregation drugs in the week leading up to admission.

On the day of admission, the patient's chest tightness worsened significantly, prompting a visit to the emergency department. Upon arrival, the patient experienced respiratory and cardiac arrest, necessitating approximately 10 min of cardiopulmonary resuscitation to restore spontaneous heart rate. Subsequently, the patient was transferred to the emergency department of Peking University International Hospital. In this setting, the patient required norepinephrine infusion to maintain blood pressure. Pulmonary examination revealed moist rales bilaterally, while cardiac and abdominal auscultation yielded no apparent murmurs. Laboratory assessments showed elevated levels of blood creatine kinase isoenzyme, myoglobin, high-sensitivity cardiac troponin T, N-Terminal Pro-Brain Natriuretic Peptide (NT-proBNP), C-reactive protein (CRP), and white blood cell count. Additionally, the patient exhibited abnormalities in interleukin-6, procalcitonin, and urinalysis.

**Figure 1:**
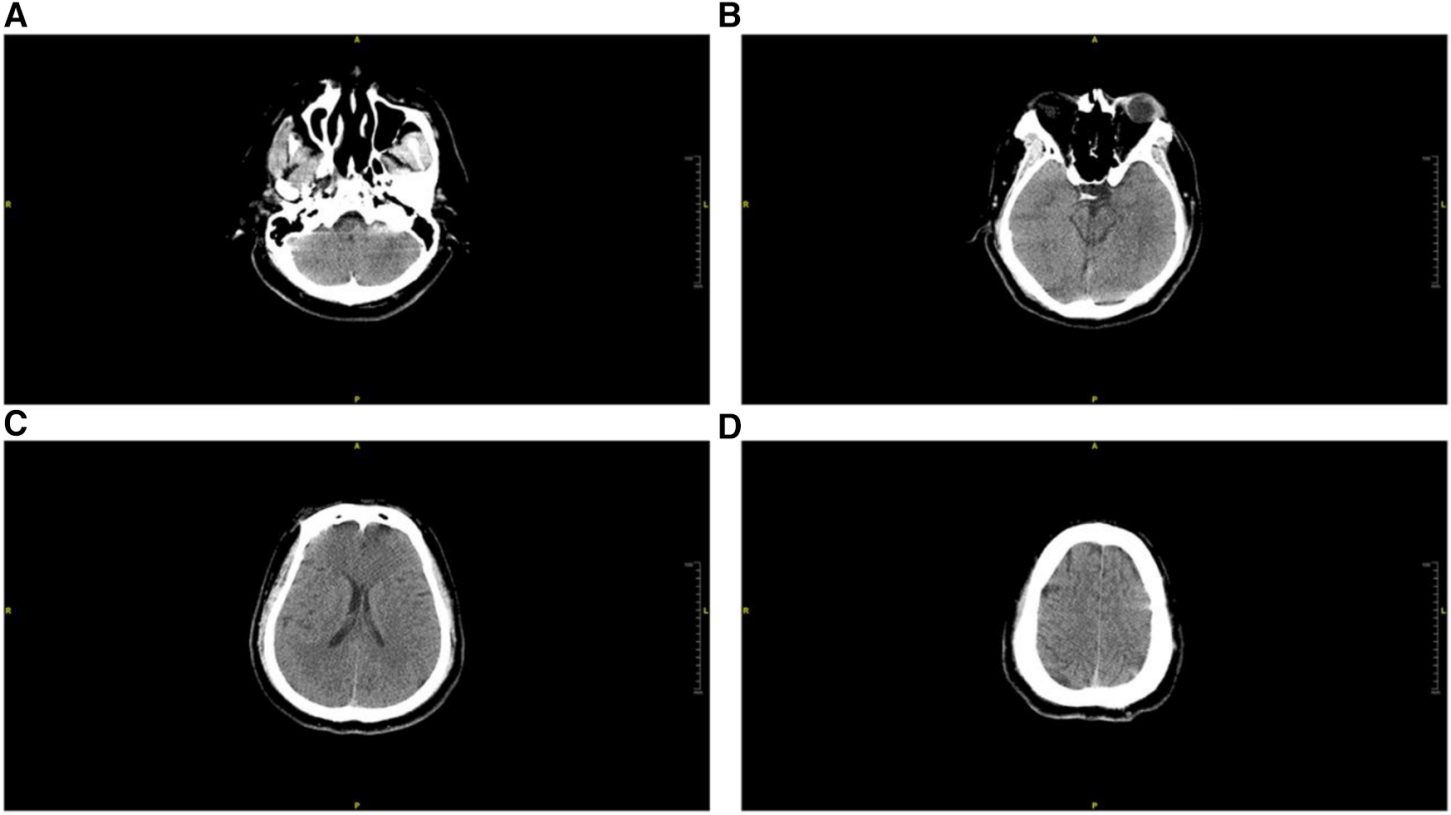
Head CT scan conducted in the emergency department during admission. (**A**) Cross-sectional view of the petrous part of the temporal bone, (**B**) cross-sectional view of the pons, (**C**) coronal section of the radiata, (**D**) superior section of the frontal lobe. There were no discernible brain lesions observed in any of the examined perspectives.

**Figure 2:**
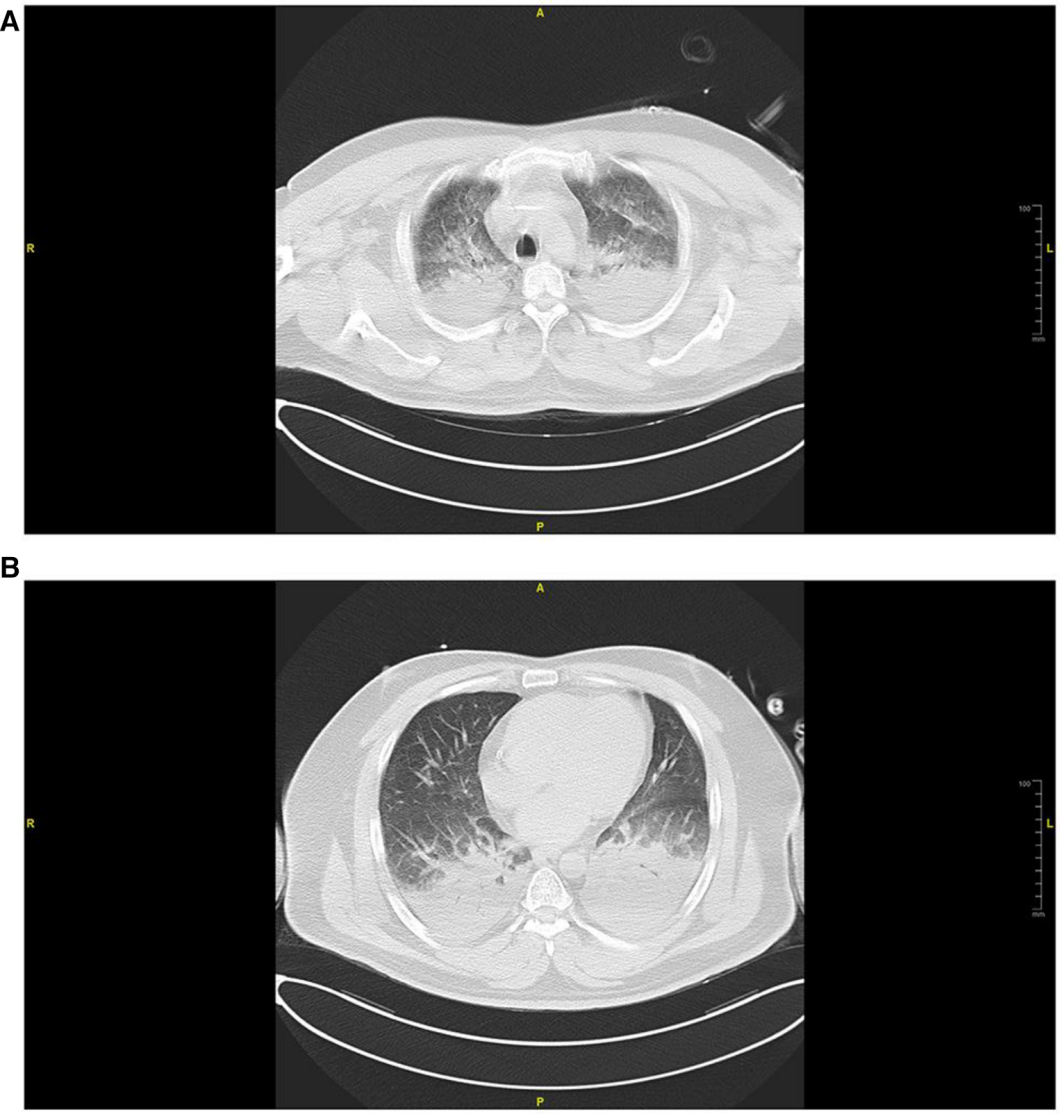
Chest CT scan conducted in the emergency room during admission. (**A**) Cross-sectional view of the aortic arch, (**B**) cross-sectional view of the mitral valve. Widespread pulmonary edema was noted bilaterally in both lungs.

Imaging studies, including head computed tomography (CT) (Figure 1), chest CT (Figure 2), and echocardiography, revealed no significant abnormalities in the head but indicated multiple infiltrates and partial consolidation in both lungs. Echocardiography showed left atrial enlargement, left ventricular ejection fraction of 39%, left ventricular diffuse hypokinesis, and no signs of pulmonary hypertension. Electrocardiography displayed Q waves in leads II, III, and aVF, along with ST segment depression and T wave abnormalities in leads V1–V6. Based on these findings, the patient was diagnosed with cardiogenic shock and subsequently admitted to the intensive care unit (ICU).

Upon admission to the ICU, the patient exhibited a progressive deterioration in clinical status, characterized by increasing rales in both lungs, elevated lactic acid levels, and decreased blood oxygen saturation. Concurrently, the administration of norepinephrine was increased to 2.0 µg/min/kg to sustain a mean arterial pressure of 65 mmHg. Echocardiography revealed a further reduction in left ventricular ejection fraction to 26%, indicating worsening cardiogenic shock. To address this exacerbation and ensure systemic tissue perfusion and oxygenation, VA-ECMO was initiated via femoral artery and vein cannulation. A 21F drainage tube and a 19F perfusion tube were inserted, and a 6F sheath was placed in the superficial femoral artery to establish distal limb perfusion and prevent ischemic necrosis.

**Figure 3:**
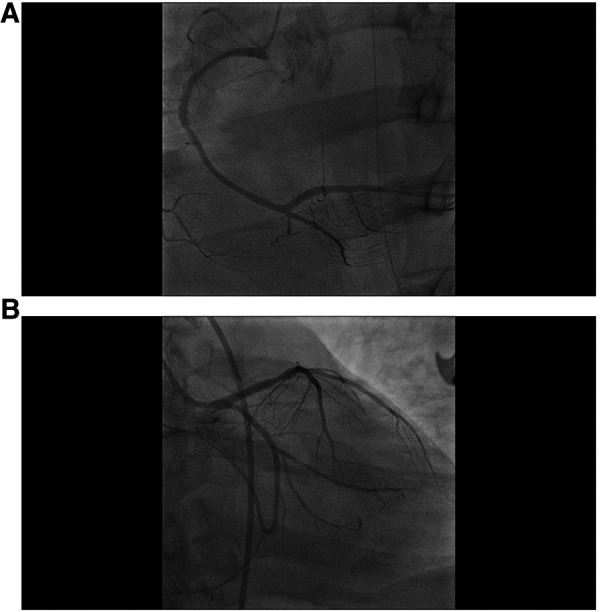
Coronary angiography results after the establishment of ECMO. (**A**) No apparent stenosis is evident in the angiography of the right coronary artery, (**B**) no apparent stenosis is observed in the angiography of the left coronary artery.

The ECMO procedure was successful, with initial settings at 3,830 rpm, a flow rate of 4.5 L/min, and a water temperature of 36.0°C. Subsequently, the mean arterial pressure was maintained at 70–80 mmHg with a reduction in norepinephrine dosage. Coronary angiography, conducted under ECMO protection, revealed no significant coronary stenosis or occlusive lesions, and all branch vessels demonstrated TIMI flow grade III (refer to Figure 3). Following the procedure, the patient was returned to the ICU without additional coronary interventions.

**Figure 4:**
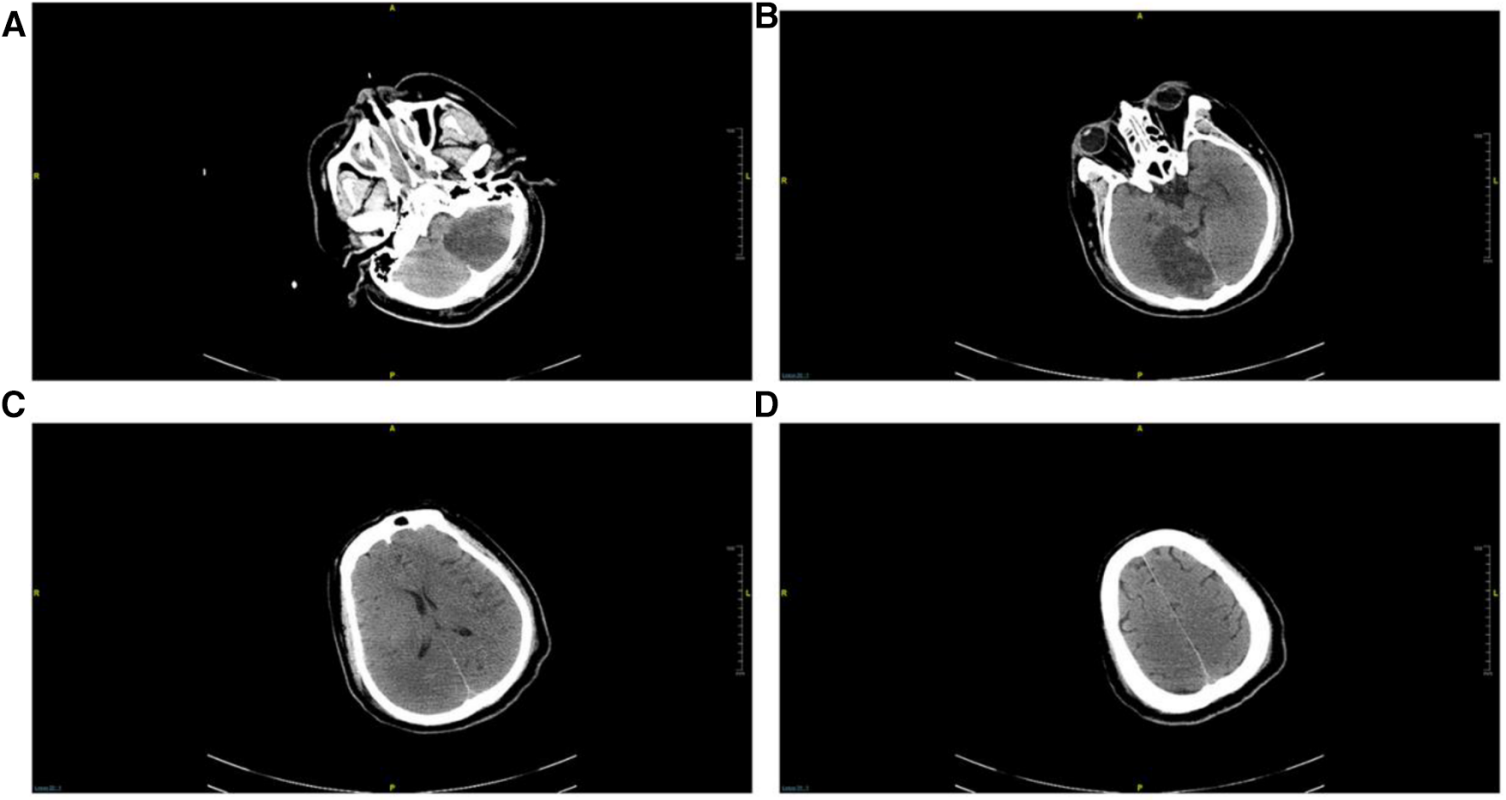
Repeated head CT on the 4th day after admission. (**A**) Horizontal cross-section of the petrous part of the temporal bone, (**B**) horizontal cross-section of the pons, (**C**) coronal radiata section, (**D**) superior cross-section of the frontal lobe. Ischemic lesions were identified in the left cerebellum and right occipital lobe, while no discernible lesions were detected in the remaining regions.

Post-transfer to the ICU, the patient developed oliguria, prompting bedside continuous renal replacement therapy (CRRT). Considering the possibility of ischemic-hypoxic encephalopathy following cardiac arrest, mannitol was administered for dehydration. CRRT was discontinued on the third day after admission. A subsequent CT scan performed on the fourth day with ECMO support, indicated marked improvement in bilateral pulmonary edema and identified denser lesions in the left cerebellar hemisphere and right occipital lobe compared to the initial emergency department CT (see Figure 4).

**Figure 5:**
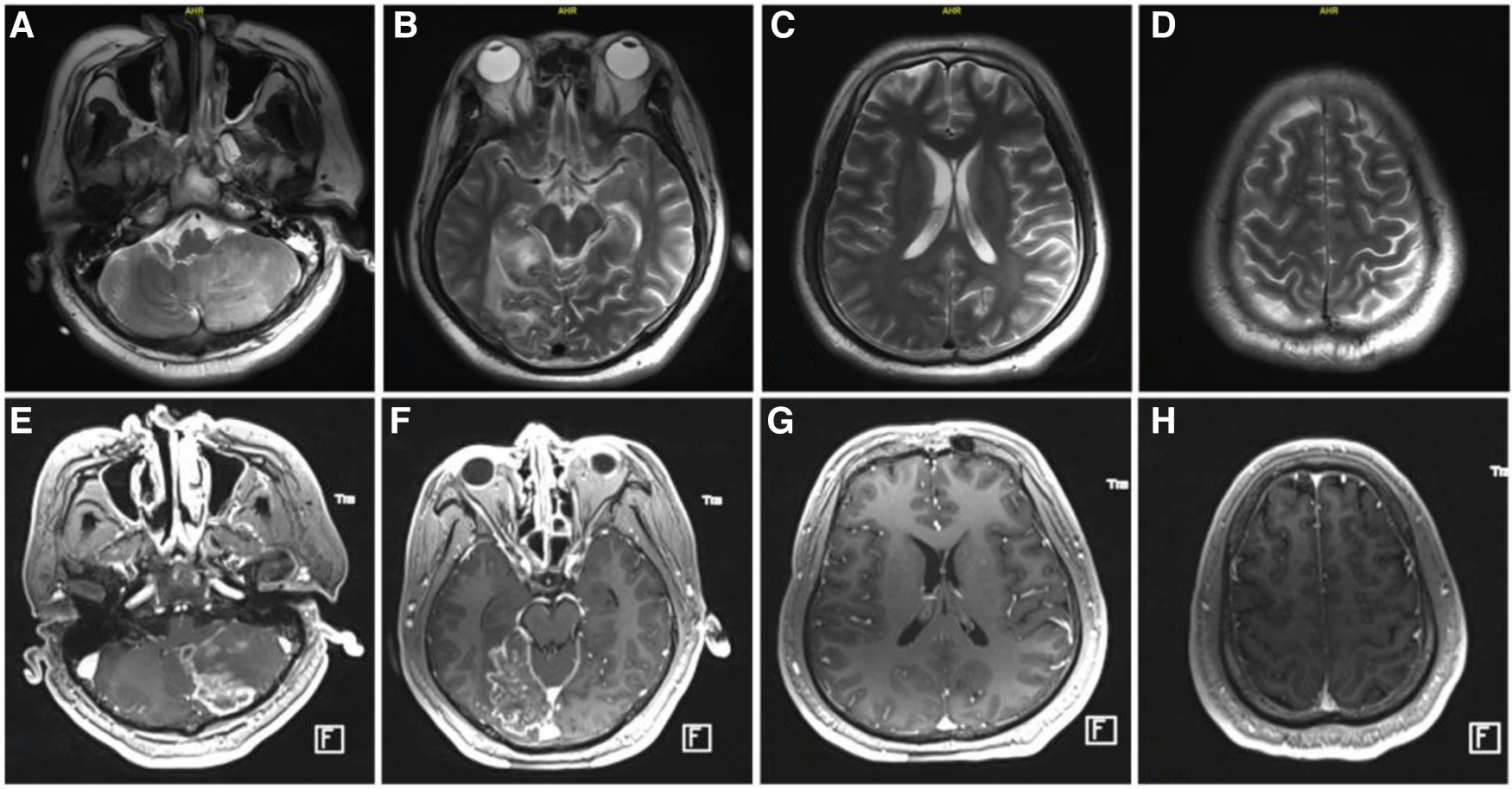
Cranial MRI at day 11 after admission. (**A**–**D**), MRI plain scan; (**E**–**H**), enhanced MRI.

By the fifth day of admission, the patient's cardiac function had recovered, achieving hemodynamic stability, and the norepinephrine dosage decreased to 0.1 µg/min/kg, while lactate levels normalized. Following a successful pump-controlled reflux test, ECMO was gradually discontinued. Post-weaning, the patient's wound healed without complications, and distal limb ischemia and necrosis were absent. On the eleventh day of admission, a cranial magnetic resonance imaging examination in the operating room (Figure 5), with ventilator support, revealed no apparent brainstem lesions, with results consistent with earlier cranial CT findings.

During this phase, following the discontinuation of sedative medication, the patient exhibited limb tremors accompanied by an elevation in heart rate and blood pressure. A transcranial Doppler examination revealed no significant impairment in blood flow to other cerebral regions. The electroencephalogram indicated diffuse reductions in the electrical amplitudes of brain waves, with no discernible abnormalities in somatosensory evoked potentials of both upper limbs and brainstem auditory evoked potentials. Consequently, the observed symptoms were attributed to paroxysmal sympathetic hyperactivity, successfully managed through the administration of β-blockers, baclofen, clonazepam, and other medications.

Regarding anti-infection management, bilateral lung imaging displayed consolidation and extensive ground-glass changes. While pulmonary edema was considered more likely, the possibility of infection-induced acute respiratory distress syndrome could not be ruled out. Therefore, a comprehensive anti-infective treatment was initiated with tienam, vancomycin, micafungin, and ganciclovir to cover a broad spectrum of potential pathogens. Blood and sputum specimens were subjected to second-generation gene sequencing, revealing 5 sequences of Klebsiella pneumoniae in the blood, 133,088 sequences of Streptococcus pneumoniae, and 2,682 sequences of K. pneumoniae in the sputum. No other pathogenic microorganisms or RNA viruses were detected. Upon receipt of bacterial results, low-level antibiotics moxifloxacin and piperacillin-tazobactam were administered on the 3rd day of admission. However, on the 10th day, sputum cultures indicated Carbapenem-resistant Enterobacteriaceae (CRE), leading to a modification in antibiotics to ceftazidime-avibactam and tigecycline on the 11th day. A tracheotomy was performed on the 11th day, allowing intermittent removal of the ventilator for respiratory function exercises.

Ultimately, on the 19th day following admission, the patient was relocated to a rehabilitation facility to undergo hyperbaric oxygen therapy. Subsequent to nearly 2 months of rehabilitation intervention, the patient exhibited significant recovery, with an improvement in the Glasgow score from E2VTM2 at the time of discharge to E4VTM5.

## Discussion

Cerebrovascular disease was initially documented by Byer et al. (6) in 1947 as a contributor to myocardial injury and tardive dyskinesia. Subsequent to this discovery, a growing body of both basic and clinical evidence has substantiated a discernible causal association between brain injury and cardiac dysfunction. Termed CCS, this relationship denotes secondary heart damage induced by various intracranial pathologies, encompassing acute cerebrovascular disease, intracranial inflammation, trauma, tumors, and intracranial hypertension. Such conditions impact cardiac function, primarily manifesting through changes in electrocardiograms, cardiac arrhythmias, elevated cardiac enzymes, and other related indicators. In severe instances, this syndrome may lead to cardiac arrest and, ultimately, mortality (1, 2).

Acute encephalopathy, such as cerebral hemorrhage, acute ischemic stroke (AIS), subarachnoid hemorrhage, and brain trauma, can be correlated with CCS. The reported incidence of CCS secondary to craniocerebral disease varies, ranging from 62% to approximately 90%, significantly impacting patient prognosis. AIS, the most prevalent brain disease, has witnessed cardiac complications emerge as a significant contributor to mortality in individuals affected by AIS. The prevalence of AIS accompanied by CCS is approximately 72.1% (7), rising to 80.2% (8) in severe cases. Studies indicate that 4.1% of patients with AIS succumb to heart disease, with 19% experiencing serious adverse cardiac events (9). In cases of CCS secondary to acute cerebral infarction, causes of death encompass primary brain tissue diseases, arrhythmia, and heart failure. Following the onset of heart failure or arrhythmia in patients with CCS, a reduction in cardiac function and ejection capacity ensues, leading to compromised cerebral perfusion, exacerbation of the primary disease, and an adverse impact on prognoses (10).

The prevailing diagnostic criteria for CCS consist of the Mayo Clinic diagnostic criteria (11). These criteria define a clinical syndrome characterized by hypokinesia of the mid-left ventricular wall following a stroke, contingent upon the exclusion of myocardial damage and dysfunction resulting from cardiac or other systemic diseases. This hypokinesia may involve the apex, and there may be abnormalities in ventricular wall motion, which fully recover during subsequent disease processes, with or without apex involvement.

There are five primary clinical manifestations of CCS: (1) Asymptomatic ischemic and non-ischemic acute myocardial injury, typically presenting with elevated troponin levels; (2) acute myocardial infarction subsequent to stroke; (3) left ventricular functional disorders, encompassing heart failure and Takotsubo syndrome; (4) alterations in electrocardiogram patterns and the onset of arrhythmias, such as atrial fibrillation following a stroke; and (5) neurogenic cardiac sudden death following a stroke (12).

The patient experienced respiratory and cardiac arrest alongside circulatory failure and shock. Following the exclusion of other shock types and cardiogenic shock from alternative causes, the diagnosis pointed towards refractory cardiogenic shock attributed to CCS. This diagnosis was established through a comprehensive assessment: despite confirming a pulmonary infection, initial and emergency room procalcitonin and C-reactive protein levels remained insignificantly elevated, ruling out septic shock. Additionally, echocardiography revealed no apparent signs of pulmonary hypertension, thus excluding the possibility of obstructive shock from acute pulmonary embolism. Furthermore, the absence of significant murmurs, aortic dissection on ultrasound, and the exclusion of myocardial ischemic disease through coronary arteriography strengthened the diagnosis. Considering the patient's extensive wet rales, glass-like shadows, solid lesions on chest CT, and left ventricular diffuse wall motion hypokinesia, typical manifestations of cardiogenic shock, coupled with subsequent cranial CT and magnetic resonance findings, the patient's acute heart failure and cardiogenic shock were attributed to CCS stemming from cerebral infarction. Based on clinical analysis, the patient met the criteria for the fifth type of CCS, necessitating prompt emergency intervention to maintain vital signs.

The etiology of CCS remains ambiguous, with primary speculation focusing on the following factors: (1) Impaired central regulation: the cardiovascular system was regulated by a complex neural network, which consists of cerebral cortex (cingulate cortex, prefrontal cortex, insular cortex), subcortical forebrain region (amygdala) and brainstem, as well as unipolar neurons as transmitting fibers, cholinergic and adrenergic neurons as efferent fibers (13, 14). When the brain was damaged, it could directly affect the heart function. (2) Neurohumoral factors (15, 16), the mechanism may be that catecholamines induce calcium overload, oxidative stress, cardiomyocyte apoptosis, cardiac hypertrophy, myocardial interstitial fibrosis and myocardial inflammation (17). At the same time, catecholamines can also cause coronary microcirculation dysfunction and exert direct toxic effects to damage the myocardium (18). In addition, cortisol also promotes the occurrence of CCS by participating in the synthesis of catecholamines (19). (3) Inflammatory transmitters or/and cytokines (20, 21), when brain injury occurred, the level of inflammatory mediators in the circulation will change and is associated with myocardial injury (22–26). In addition, the stress response triggered by AIS can damage the intestinal barrier, resulting in the dysbiosis and translocation of intestinal flora. Intestinal inflammatory factors will also enter the circulatory system, enhance the systemic inflammatory response, and then damage the heart (27, 28). (4) There is a common basis of vascular disease (29). Cerebrovascular disease and cardiovascular disease have a common pathological basis, such as hypertension, diabetes, hyperlipidemia, etc. (5). Neuropeptides: neuropeptide Y is the third type of autonomic neurotransmitter newly discovered in recent years. It belongs to non adrenergic non cholinergic peptidergic nerves, and its role in the occurrence and development of CCS has been paid more and more attention by scholars (30). (6) Others: damaged or dead neuronal cells will secrete soluble toxic factors in the early stage, and transmit death signals through the circulatory system to regulate cardiomyocyte death (31); Low expression of microRNA-126 in AIS plays an important role in cardiac dysfunction (32–34); In addition, electrolyte disturbance can also lead to cardiac dysfunction and arrhythmia.

In the context of treatment, addressing reversible cardiac dysfunction, such as CCS, is crucial, particularly in cases where cardiogenic shock poses a life-threatening risk. The foremost priority is to effectively sustain the patient's circulatory and respiratory functions, thereby preserving their life. This intervention provides a window of opportunity for subsequent cardiac recovery and facilitates the administration of further treatment, including rehabilitation therapy.

While ECMO has been utilized successfully in cases of neurogenic pulmonary edema secondary to craniocerebral diseases and hemorrhagic conditions, its application in CCS has been less extensively documented (4, 5). In this study, we present a successful case where ECMO was used as an adjunctive treatment for circulatory failure resulting from CCS.

The ECMO team responded swiftly to establish circulatory support, preventing the patient's demise and creating a critical time window for subsequent treatment. Ultimately, the patient was successfully transferred to a rehabilitation hospital for further neurological rehabilitation. This case underscores the life-saving impact of timely ECMO support in critical situations since the establishment of the ECMO team in the hospital.

Moreover, throughout the treatment, implementing interventions such as sedation, management of muscle fibrillation, administration of mannitol, and regulation of the ECMO tank's temperature proved to be successful in maintaining effective control over the patient's intracranial pressure without exacerbating neurological damage. Previous studies have indicated the viability and efficacy of intravascular cooling in individuals with refractory high intracranial pressure (35). The presence of a temperature control system within the ECMO system itself facilitates its utilization as an intravascular cooling method, contributing to the protection of the patient's neurological function to some extent.

## Conclusion

In instances where cardiogenic shock arises as a consequence of an ischemic stroke, resulting in CCS, the utilization of VA-ECMO may serve as a viable method of circulatory assistance. This approach is capable of sustaining systemic tissue perfusion in patients, safeguarding neurological function, and allowing a critical window for additional diagnostic measures and interventions. Consequently, this intervention holds the potential to enhance the overall survival rate of affected individuals.

## Data Availability

The original contributions presented in the study are included in the article/Supplementary Material, further inquiries can be directed to the corresponding authors.
